# Cytosolic DNA sensing by cGAS: regulation, function, and human diseases

**DOI:** 10.1038/s41392-021-00554-y

**Published:** 2021-04-30

**Authors:** Le Yu, Pengda Liu

**Affiliations:** 1 Lineberger Comprehensive Cancer Center, The University of North Carolina at Chapel Hill, Chapel Hill, NC USA; 2 Department of Biochemistry and Biophysics, The University of North Carolina at Chapel Hill, Chapel Hill, NC USA

**Keywords:** Innate immunity, Cancer microenvironment

## Abstract

Sensing invasive cytosolic DNA is an integral component of innate immunity. cGAS was identified in 2013 as the major cytosolic DNA sensor that binds dsDNA to catalyze the synthesis of a special asymmetric cyclic-dinucleotide, 2′3′-cGAMP, as the secondary messenger to bind and activate STING for subsequent production of type I interferons and other immune-modulatory genes. Hyperactivation of cGAS signaling contributes to autoimmune diseases but serves as an adjuvant for anticancer immune therapy. On the other hand, inactivation of cGAS signaling causes deficiency to sense and clear the viral and bacterial infection and creates a tumor-prone immune microenvironment to facilitate tumor evasion of immune surveillance. Thus, cGAS activation is tightly controlled. In this review, we summarize up-to-date multilayers of regulatory mechanisms governing cGAS activation, including cGAS pre- and post-translational regulations, cGAS-binding proteins, and additional cGAS regulators such as ions and small molecules. We will also reveal the pathophysiological function of cGAS and its product cGAMP in human diseases. We hope to provide an up-to-date review for recent research advances of cGAS biology and cGAS-targeted therapies for human diseases.

## Introduction

Germline-encoded pattern-recognition receptors (PRRs) are key players for human innate immunity. Depending on the source of products, PRRs are divided into two groups, including pathogen-associated molecular patterns (PAMPs) and danger-associated molecular patterns (DAMPs).^1–3^ Pathogen-derived nucleic acids including DNA and RNA are detected by PRRs that subsequently trigger downstream innate-immune responses.^2–6^ Over the past two decades, a variety of PRRs have been identified, including Toll-like receptors (TLRs), C-type lectin-like receptors (CLRs), retinoic acid-inducible gene I–like receptors (RLRs), NOD-like receptors (NLRs), and the absent in melanoma 2 (AIM2)-like receptors (ALRs).^7,8^

**Fig. 1:**
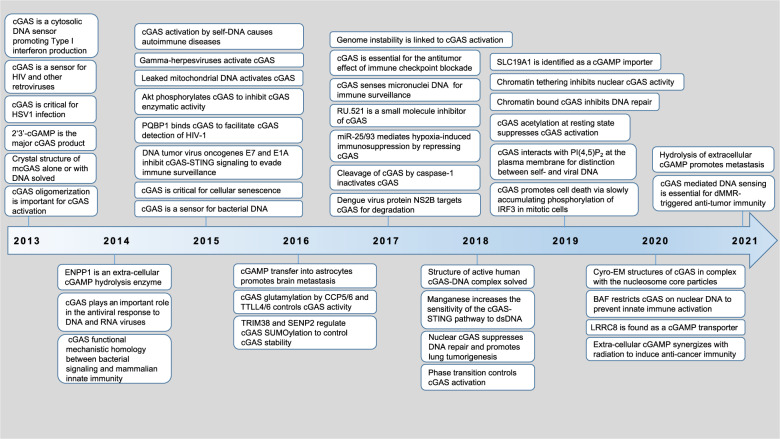
A timeline for discoveries of cGAS regulation and function. Due to a large amount of work on this topic in the past 8 years, we cannot include all major findings in this time table and we sincerely apologize for colleagues whose important work are not mentioned in this figure due to space constraints

TLRs and CLRs are membrane-associated receptors, while RLRs, NLRs, and ALRs are cytosolic nucleotide sensors. Cytosolic DNA derived from either pathogens (non-self-DNA, including viral and bacterial DNA) or host genome (self-DNA, including damaged mitochondrial DNA (mtDNA), leaked/damaged nuclear DNA from chromosome instability (CIN), cytosolic DNA in micronuclei and from cell debris), are powerful activators for the innate-immune system. There are four major ALRs all belonging to the PYHIM family members identified in human, including AIM2,^9–11^ interferon-inducible protein 16 (IFI16),^12^ interferon-inducible protein X (IFIX),^13^ and myeloid nuclear differentiation antigen (MNDA).^14^ In addition, other candidates have also been proposed to sense cytosolic DNA, including DNA-dependent activator of IRFs (DAI),^15^ LRR binding FLII interacting protein 1 (LRRFIP1),^16^ RNA polymerase III,^17,18^ Ku heterodimers (Ku70 and Ku80),^19,20^ DExD/H box helicases (DDX41),^21^ meiotic recombinations 11 homolog A (MRE11),^22^ and others. Notably, distinct from other DNA sensors that stimulate interferon production, AIM2 activation in macrophages triggers the formation of a multiprotein complex named inflammasome, leading to activation of the protease procaspase 1 that cleaves pro-IL-1β and pro-IL-18 in triggering proptosis,^23^ a process antagonized by p202.^24^ These PRRs are cell-type or DNA-sequence specific,^25,26^ thus excluding their function as a universal cytosolic DNA sensor. In 2013, the cyclic GMP–AMP (cGAMP) synthase (cGAS) was identified as one of the most important cytosolic DNA sensors,^27^ given that cGAS recognizes and responds to cytosolic DNA in a DNA-sequence-independent but DNA length-dependent manner in various cell types. Since its discovery, it quickly draws extensive attention from researchers. Within only 8 years, our understanding of cGAS structure, regulation, and function in human diseases has been significantly advanced due to contributions from many intriguing studies. These include but not limited to the identification of cGAS as an essential cytosolic DNA sensor for DNA viruses, RNA viruses, damaged mitochondrial and genomic DNA, illustrations of cGAS activation mechanisms by both structural and biochemical analyses, regulatory mechanisms controlling cGAS activation by cGAS modifications, binding partners and ions, pathophysiological roles of cGAS in biological processes and human diseases, nuclear cGAS function in regulating DNA damage repair and tethering with chromatin, as well as regulations and function of the cGAS enzymatic product cGAMP (Fig. 1).

**Fig. 2:**
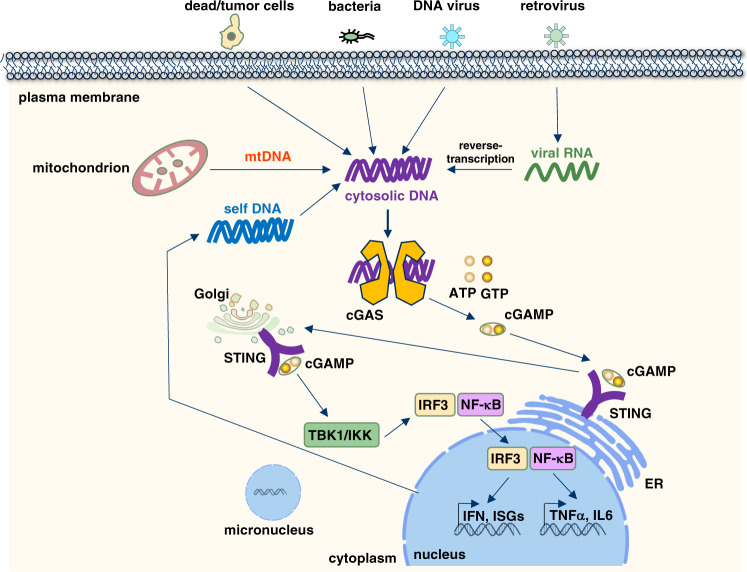
The cGAS–STING signaling pathway senses cytosolic DNA derived from either viral/bacterial infection or self-DNA. DNA is a pathogen-associated molecular pattern when delivered to the host cytoplasm by viral or microbial infection, and a danger-associated molecular pattern when leaked into the cytoplasm from damaged mitochondria or nucleus. cGAS is the cytosolic DNA sensor that recognizes and binds cytosolic DNA in a DNA-sequence-independent manner that subsequently triggers cGAS dimerization and production of a special dinucleotide messenger, 2′3′-cGAMP from ATP and GTP. 2′3′-cGAMP binds STING localized on ER, through trafficking to Golgi to recruit and activate IKK and TBK1. TBK1 phosphorylates STING, which in turn recruits IRF3 for phosphorylation by TBK1. Phosphorylated IRF3 dimerizes and enters the nucleus, where it cooperates with NF-κB signaling to turn on transcription of type I IFNs and other immunomodulatory genes

cGAS (also known as C6orf150, or male abnormal 21 domain containing 1 (MAB21D1)) is located on chromosome 6q13 and encodes a protein with 522 amino acids in human. Mechanistically, cGAS recognizes cytosolic dsDNA in a DNA length-dependent but DNA-sequence-independent manner. Interestingly, oxidized self-DNA (8-OHG), although resistant to TREX-1 degradation, could still be recognized by cGAS to promote cGAS activation and innate-immune recognition.^28^ Short dsDNA (<20 bp) binds but fails to activate cGAS due to its inability to induce cGAS dimerization.^29,30^ Longer dsDNA (>20 bp) activates cGAS through promoting cGAS dimerization by forming a 2:2 DNA/cGAS complex,^31^ allowing for rearrangement of the cGAS catalytic pocket for subsequent binding of cGAS substrates adenosine triphosphate (ATP) and guanosine triphosphate (GTP)^32^ to induce the synthesis of 2′3′-cGAMP.^33,34^ cGAMP then binds STING (also known as TMEM173, MPYS, ERIS, and MITA) on ER membrane^35–37^ to further recruit TBK1 that facilitates IRF3 phosphorylation and subsequent interferon β (IFNβ) production to trigger inflammation, adaptive immunity^38–41^ and expression of other co-regulated genes (Fig. 2).^42^ STING as an endoplasmic reticulum (ER) localized protein is composed of four N-terminal transmembrane helices and one globular C-terminal cytosolic domain (CTD).^38^ STING activation is regulated by a number of candidate DNA sensors, including cGAS, IFI16, DDX41, MRE11, and Lsm14A. STING recruits TBK1 to phosphorylate STING on Ser366 residue that further recruits IRF3, where TBK1 phosphorylates IRF3 for its nuclear translocation, dimerization, and activation, which is necessary for IFNβ transcription.^34^

In addition, cGAS dimmers form higher orders of oligomers^31^ and undergo liquid phase separation^43^ that significantly enrich local concentrations of cGAS/DNA to further boost cGAS activation. A recent study reveals that cGAS phase transition precludes TREX-1 from degrading dsDNA, thus sustaining DNA-induced cGAS activation.^44^ Interestingly, although ssDNA and dsRNA can bind cGAS, they fail to rearrange the cGAS catalytic pocket for cGAS activation. The cGAS protein is composed of an unstructured and not well-conserved N-terminus (amino acid residues 1–160) and a highly conserved C terminus (161–522).^45^ The cGAS N-terminal fragment is highly disordered with a number of K/R residues that have been predicted to bind DNA, albeit the detailed mechanism(s) remains to be elucidated.^46^ This unstructured N-terminus may also play a role for cGAS plasma membrane attachment to restrain cGAS activation.^47^ The cGAS C-terminal domain contains two strongly conserved motifs, including a nucleotidyltransferase (NTase) core domain (160–330) and a Mab21 domain with zinc-ribbon insertion (213–513).^31,48^ The NTase domain is indispensable for cGAS enzyme activity.^27^ The conserved ZnF motif is vital for DNA binding, enzymatic activity, and downstream innate-immune signaling activation. Notably, the cGAS C-terminal domain contains a strong DNA-binding site A, a weaker DNA-binding site B,^31,48^ and an additional DNA-binding site C^49^ that facilitates cGAS activation and phase transition.

cGAS shares significant sequence similarity to the RNA sensor oligoadenylate synthase 1 (OAS1), indicating that cGAS and OAS1 may be derived from an evolutionarily related family of enzymes involved in host immune response.^50,51^ Interestingly, analyses of cGAS homologs in different species suggest that cGAS protein sequence is ancestral, and its cytosolic DNA sensor function might be conserved during evolution.^52^ Given to the unique role of cGAS in sensing cytosolic DNA regardless of its origin (including both exogenous and endogenous DNA) and sequence, cGAS exerts remarkably diverse regulatory functions in a variety of cellular progresses including DNA damage response, tissue fibrosis, senescence, inflammation, cell death, autophagy, and tumorigenesis.^53–64^ Hyperactivation of cGAS/STING signaling plays an indispensable role in the development of autoimmune diseases such as Aicardi–Goutieres Syndrome (AGS) and systemic lupus erythematosus (SLE),^65–67^ while on the other hand helps to establish an immune-friendly microenvironment by promoting T-cell infiltrations into tumors.^68^ In this scenario, a tightly controlled and balanced cGAS activation is necessary to maintain proper cell physiology and function. In this review, we summarize up-to-date knowledge for cGAS regulatory mechanisms, including cGAS expression regulations at DNA, RNA, and protein levels, cGAS activation controls by protein post-translational modification and binding proteins, as well as ions and small molecules. We will also review cGAS function in human diseases and current knowledge and trials in targeting cGAS, or its enzymatic product, 2′3′-cGAMP for disease treatment.

## Regulatory mechanisms for cGAS activity control

### *cGAS* gene alternations are rarely observed in human diseases

Although cGAS plays critical pathophysiological roles in autoimmune diseases, aging, and cancer, the *cGAS* gene has not been reported to be amplified/mutated/deleted in these human diseases. Querying TCGA datasets led us to find that the *cGAS* gene is mutated (13 in 20 cases) in SCLC (small cell lung cancer) patients and deleted (23 in 237 cases) in metastatic breast cancer patients (the MBC project). Thus, it is plausible that *cGAS* gene alternations may contribute to cancer phenotypes; however, more in-depth investigations are warranted to examine this concept. These observations suggest that alternations in *cGAS* gene may not be a major route through which cGAS activity is deregulated in human diseases.

### Transcriptional and post-transcriptional regulations of cGAS expression

The region (−414 to +76) next to the transcription start site (TSS) in the *cGAS* gene was found to be critical as a promoter for maintaining cGAS transcription.^69^ Mutating Sp1 and CREB-binding motifs in this region led to reduced cGAS transcription,^69^ suggesting that both transcription factors govern cGAS transcription. In addition, an epigenetic cofactor NCOA3 was observed to maintain basal cGAS expression,^70^ while the identity of responsible transcription factors remains to be determined. In microglia, HDAC3, as a member of histone deacetylases, was found to be crucial for cGAS transcription by deacetylating p65 to enhance p65 association with cGAS promoter to transcriptionally potentiate cGAS expression.^71^

MicroRNAs (miRNAs) downregulate their target gene expression at post-transcriptional levels through binding the 3′ untranslated regions (3′UTRs) of the target messenger RNA (mRNA).^70^ It has been documented that a number of miRNAs are involved in DNA-sensing-related immune defense^72,73^ and are pivotal for presenting antigens and secreting immuno-cytokines. Hypoxia-responsive miRNAs including miR-93 and miR-25 were reported to remarkably downregulate cGAS expression in the immunosuppressive tumor microenvironment (TME) through targeting NCOA3 for suppression.^70^ Thus elevated expression of miR-93/25 observed in breast cancer facilitates tumor evasion from immune surveillance and destruction partially through downregulating cGAS expression. In addition to miRNAs, an HSV-1 tegument protein UL41 was also observed to utilize its endoribonuclease activity to degrade cGAS mRNA, through which UL41 negatively regulates DNA sensing to facilitate HSV-1 infection by escaping from immune surveillance.^74^

### Post-translational regulations of cGAS activation

In addition to genetic and transcriptional controls, cGAS activity is also modulated at post-translational levels. Prior work identified multiple regulatory mechanisms governing cGAS activation, including regulations by post-translational modifications and binding proteins. In this section, we summarize major findings in these aspects. Notably, given that both human and mouse cGAS molecules have been used in these studies, and <60% cGAS protein sequence is shared between these two species, to clarify the exact amino acid(s) being modified, in this section we will label human and mouse cGAS proteins as hcGAS and mcGAS, respectively.

#### Regulation of cGAS activation by cGAS post-translational modifications

##### Control of cGAS activation by ubiquitin

As a reversible post-translational modification, protein ubiquitination or deubiquitination plays an indispensable and evolutionarily conserved function in both eukaryotes and prokaryotes^75,76^ in regulating a diversity of cellular processes, including degradation of unwanted proteins, cell cycle progression, DNA damage response, vesicle transport, endocytosis, signal transduction, and others.^77,78^ Protein ubiquitination is largely carried out by a cascade of enzymatic reactions governed by E1 ubiquitin-activating enzymes, E2 ubiquitin-conjugating enzymes, and E3 ubiquitin ligases. Among all these three categories of enzymes, it is the E3 ligase that determines ubiquitin substrate specificity. There are ~600 E3 ubiquitin ligases encoded in human genome that fall into three families: RING (really interesting new gene), HECT (homologous to E6AP carboxyl terminus), and RBR (RING-between-RING).^78^ Poly-ubiquitin chains, established by E3 ligase(s), can be removed by deubiquitinases (DUBs). E3 ligases and DUBs are diverse in structure and function with a myriad of distinct mechanistic features. Compared with many E3 ligases, there are only ~100 DUBs, suggesting that DUBs are less selective towards substrates. DUBs are currently classified into six families, including ubiquitin C-terminal hydrolases (UCHs), ubiquitin-specific proteases (USPs), ovarian tumor proteases (OTUs), Josephins, JAB1/MPN/Mov34 metalloenzymes (JAMMs), and motif interacting with Ub-containing novel DUB family (MINDYs).^79^ Although these DUBs are structurally unrelated, they all interact with a common hydrophobic patch on ubiquitin. Emerging evidence reveals roles of dysregulated E3 and DUB signaling in contributing to human diseases including cancer,^80^ immune diseases,^81,82^ brain diseases,^83^ and others. Agents modulating DUB activities^84^ or impeding E3-ligase interactions with substrates^85^ have been developed and tested in clinics, and PROTAC (proteolysis-targeting chimera)^86^ has emerged as a novel and powerful tool in this regard as a promising treatment direction utilizing E3-ligase-mediated protein-degradation processes. It is not surprising that cGAS is also under regulation by ubiquitin modifications.

**Fig. 3:**
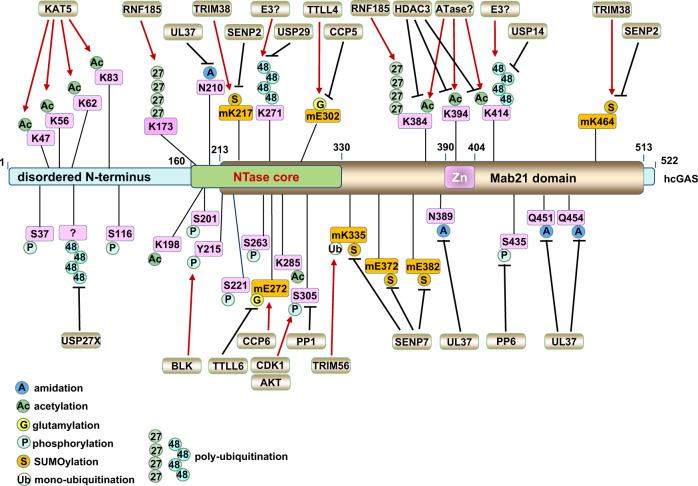
Regulations of cGAS activation by cGAS post-translational modifications. cGAS is dynamically regulated by various post-translational modifications in responding to DNA insults, such as monoubiquitination, polyubiquitination, SUMOylation, glutamylation, phosphorylation, acetylation, and deamidation in cells. This figure illustrates up-to-date reported post-translational modifications occurring on cGAS proteins, including the modified residues and modifying enzymes. Please note that all human cGAS residues are labeled in pink while all mouse cGAS residues are labeled in orange with “m” inserted in front of the residue

The presence of seven lysine residues in each ubiquitin molecule assigns the possibility for the formation of diverse poly-ubiquitin chain linkages with distinct topologies and physiological functions.^87^ cGAS undergoes ubiquitin-mediated modifications including monoubiquitination and polyubiquitination that differentially modulate cGAS activation and function (Fig. 3).

### Monoubiquitination of cGAS promotes cGAS activation

Two E3 ubiquitin ligases including Tripartite-motif containing (TRIM) E3 ligases TRIM56 and TRIM41 (RINCK) have been found to be responsible for cGAS monoubiquitination. TRIM56 was identified as a cGAS-binding protein in a proteomics study and was found to promote mcGAS monoubiquitination on Lys335,^88^ which led to enhanced cGAS dimerization, DNA binding, and cGAMP production. TIRM41 was also identified as a cGAS interactor by proteomics and shown to be able to promote cGAS monoubiquitination in biochemical assays.^89^ Although the modifying lysine residue(s) by TRIM41 remains unknown, this cGAS monoubiquitination is indispensable for cGAS activation upon DNA challenge to exert its full activities.^89^ The exact molecular details for how monoubiquitination of cGAS facilitates cGAS dimerization and DNA binding remain unclear. Although the mono-ubiquitin moiety does not directly bind DNA,^90^ given that the Lys335 residue is located to the close proximity of DNA-binding region in mcGAS (PDB 4LEZ), it is plausible that this monoubiquitination modification may help stabilize the ternary complex formed by cGAS dimers with two DNA molecules.

### cGAS polyubiquitination in controlling cGAS activation and function

In addition to monoubiquitination, cGAS is also modified by polyubiquitination. The RING finger (RNF) containing E3 ubiquitin ligase RNF185 located on ER induces an accumulation of K27-linked poly-ubiquitin chains on Lys173/Lys384 residues in mcGAS, through an interaction mediated by the RING domain of RNF185 and the C-terminal domain of cGAS. K27-linked polyubiquitination of cGAS enhanced cGAS enzymatic activity both in vitro and in cells for cGAMP synthesis.^91^ Given K27-linked polyubiquitination of STING recruits TBK1 binding^92^ and K27-linked polyubiquitination of NEMO promotes Rhbdd3 binding,^93^ we speculate if cGAS recognizes K27-linked poly-ubiquitin chains so that K27-linked polyubiquitination of cGAS promotes cGAS dimerization or oligomerization. This requires further experimental evidence to support or reject the hypothesis. Interestingly, HSV-1 infection promotes the co-localization of RNF185 and cGAS in cells,^91^ suggesting that cells may utilize K27-linked ubiquitination to promote cGAS activation responding to DNA viral infection.

TRIM14 is a member of the tripartite-motif (TRIM) E3 ubiquitin ligase family with identified roles in facilitating sensing RNA viruses as a mitochondria adaptor.^94^ Although as a noncanonical TRIM lacking a RING domain, TRIM14 was found to be able to stabilize cGAS proteins by recruiting USP14 to cleave K48-linked poly-ubiquitin chains at the hcGAS-Lys414 residue, given that K48-linked polyubiquitination of hcGAS-Lys414 primes cGAS for p62 binding that targets cGAS for lysosomal degradation.^95^ In another study, TRIM14 was reported to bind cGAS and TBK1, and bridge TBK1 binding to STAT3 to promote STAT3 phosphorylation in the synthesis of ISGs (interferon-stimulated genes) that facilitate detection and clearance of *Mycobacterium tuberculosis* infection.^96^ Interestingly, TRIM14 itself is a transcriptional target for interferon signaling,^97^ which suggests that there might be a positive regulatory loop for augmenting innate immunity. Notably, the identity of the E3 ligase(s) that targets cGAS protein for ubiquitination and degradation remains elusive to date.

### cGAS deubiquitination in controlling cGAS activation and function

Among all DUBs, 79 of them exert enzymatic activity. Members of the ubiquitin-specific peptide (USP) family of DUBs have emerged as vital molecules in regulating antiviral immunity, either as direct regulation factors of viral replication or as regulators of host nucleic acid-sensing pathways.^98^ It was reported that upon DNA virus infection, USP14 was recruited by TRIM14 to remove K48-linked ubiquitin chains at the Lys414 site in hcGAS, leading to cGAS stabilization to promote antiviral innate immunity.^95^ Similarly, USP27X^99^ and USP29^100^ have also been reported to stabilize cGAS proteins by cleaving K48-linked poly-ubiquitin chains from cGAS, and both of them serve as positive regulators in activating innate immunity to fight against DNA viral infection.

### Regulation of cGAS activation by SUMOylation

Similar to ubiquitination, protein SUMOylation can rapidly regulate protein fate and function. SUMO (small ubiquitin-like modifier) is predominantly found in the nucleus with important roles in regulating various pathophysiological processes,^101^ including DNA damage response,^102^ cancer, Huntington’s disease, Alzheimer’s disease, Parkinson’s disease^103^ and innate immunity.^104^ Previously, it was reported that the E3 ubiquitin ligase TRIM38 negatively regulates TLR-mediated immune signaling by ubiquitinating TRIF and promoting TAB2/3 degradation.^105^ On the other hand, TRIM38 was reported to positively regulate RLR-induced innate immunity by SUMOylating MDA5^106^ and RIG-I^107^ for their stabilization through antagonizing ubiquitination-mediated protein degradation. Recently, TRIM38 was also shown to be able to modulate cytosolic DNA sensing and cGAS activation. Instead of regulating cGAS ubiquitination, at the resting state or early infection stages, TRIM38 maintains SUMOylation at Lys217 and Lys464 residues in mcGAS (corresponding to Lys231 and Lys479 in hcGAS), which prevents K48-linked cGAS polyubiquitination that directs cGAS for protein destruction.^108^ Thus, through stabilizing cGAS proteins, TRIM38 ensures cGAS availability in responding to DNA viral infection. Together, these observations suggest that TRIM38 may exert function in suppressing immune response as an E3 ubiquitin ligase, while potentiate DNA or RNA virus-induced innate immunity as a SUMOylase. At later stages of viral infection, the deSUMOylating enzyme Sentrin/SUMO-specific protease (SENP) 2 removes SUMO modifications from cGAS, subsequently promoting K48-linked ubiquitination at mcGAS-Lys464 residue (corresponding to Lys479 in hcGAS) that primes cGAS for degradation by the ubiquitin–proteasome pathway to attenuate the antiviral response.^108^ On the other hand, SUMOylation of mcGAS at Lys335/Lys372/Lys382 residues by unknown enzymes suppresses DNA binding, cGAS oligomerization, and cGAS nucleotidyltransferase activity, while SENP7 alleviates SUMO-mediated suppression of cytosolic DNA sensing by removing cGAS SUMOylation and potentiates cGAS activity.^109^ Notably, increased expression of either TRIM38^108^ or SENP7^109^ has been observed in patients with SLE (systemic lupus erythematosus), further revealing the pathophysiological significance of cGAS deSUMOylation in activating cGAS governed cytosolic DNA-sensing signaling. Taken together, cGAS SUMOylation can either potentiate cGAS activation through stabilizing cGAS proteins by antagonizing cGAS ubiquitination-mediated degradation or suppress cGAS activation by impeding cGAS dimer formation or DNA binding, depending on the modified lysine residues.

### Regulation of cGAS activation by glutamylation

Protein glutamylation is a type of ATP-dependent post-translational modifications (PTM) vital for regulating bacterial and viral infection that involves the conjugation of glutamate side chains to the γ-carboxyl groups of glutamic acid residues in target proteins.^110^ Glutamylation is usually catalyzed by glutamylases and removed by carboxypeptidases.^111^ Recently, cGAS was reported to undergo glutamylation modifications. Specifically, TTLL4 (tubulin tyrosine ligase-like 4) and TTLL6 catalyzed mono-glutamylation and poly-glutamylation of mcGAS at Glu302 and Glu272 residues, respectively. TTLL4-mediated mono-glutamylation of cGAS inhibits cGAS enzymatic activity and TTLL6-governed cGAS poly-glutamylation attenuates DNA binding, both leading to reduced synthase activity of cGAS for cGAMP production.^112^ On the other hand, carboxypeptidases CCP5 and CCP6 removed mono- and poly-glutamylation modifications on mcGAS-Glu302 and Glu272, respectively, leading to cGAS activation.^112^ These findings provide additional insights into the fine-tune mechanisms for cGAS activity regulations that have been evolved during evolution to ensure cells adapt and respond to external invasive cues in an acute and regulatable manner.

### Regulation of cGAS activation by phosphorylation

Protein phosphorylation is one of the most extensively studied protein modifications in dynamically regulating protein functions in a plethora of biological processes, including cell cycle regulation, apoptosis, DNA damage response, tumorigenesis, and immunity.^113,114^ Small-molecule kinase inhibitors (SMKIs) have been widely developed and tested as promising targeted therapeutics.^115^ The first post-translational modification reported on cGAS was AKT-mediated mcGAS phosphorylation at Ser291 (corresponding to Ser305 in hcGAS).^116^ AKT-mediated cGAS phosphorylation occurs in the carboxyl-terminal enzymatic domain of cGAS and results in suppression of cGAS enzymatic activity in cGAMP synthesis to alleviate immune response upon viral infection.^116^ Given that hyperactivation of the PI3K/AKT signaling has been widely observed in human cancers^117^ and evading immune surveillance and destruction is a hallmark of cancer,^118^ it is plausible that tumors hijack AKT/cGAS signaling to inactivate cGAS in creating an immune environment favored by tumors. In addition, recently the CDK1-cyclin B kinase complex was reported to phosphorylate hcGAS at Ser305 residue (Ser291 in mcGAS) as well, which inhibits its ability to synthesize cGAMP in mitotic cells.^119^ This process can be antagonized by the protein phosphatase 1 (PP1) through dephosphorylating cGAS upon mitotic exit to enable its DNA-sensing ability.^119^ Considering that Akt activity is also controlled in a cell cycle-dependent manner with peak activity at S/G2,^120^ it is plausible that Akt and CDK1 govern cGAS phosphorylation in S and M phases, respectively, to ensure cGAS activation is properly controlled during critical cell cycle phases with DNA replication (S) and DNA separation (M) accompanied by increased chances of cGAS exposure to genomic DNA. Recent studies reported that protein phosphatase 6 (PP6) dephosphorylates mcGAS at Ser420 (Ser435 in hcGAS) to restrain its substrate-binding ability and suppress cGAMP synthesis.^121^ Moreover, Tyr215 in hcGAS has been reported to be phosphorylated by BLK (B lymphocyte kinase) that retains cGAS in the cytoplasm,^64^ where cGAS is primed for cytosolic DNA sensing. Considering that deregulation of BLK,^122^ PP1, and PP6^123^ has been reported in various cancers and other human diseases, if and how these enzymes contribute to pathological processes through regulating cGAS modifications remains an interesting question.

### Regulation of cGAS activation by acetylation

Protein acetylation occurs on lysine residues and is a result of a balanced action by acetyl-transferases and deacetylases.^124^ Acetylation events at the N-terminus of histones have been well-documented as key events for epigenetic regulation of gene transcription. hcGAS was found to be acetylated at Lys384/Lys394/Lys414 residues at resting states to restrain cGAS activation. Upon DNA challenge, cGAS was deacetylated on these sites allowing for cGAS activation.^125^ Interestingly, aspirin could directly acetylate cGAS to inhibit cGAS activation, which reveals a promise in applying aspirin in treating autoimmune diseases such as Aicardi–Goutieres syndrome (AGS).^125^ Recently, the lysine acetyl-transferase KAT5 was reported to acetylate Lys47/Lys56/Lys62/Lys83 residues located in the N-terminal unstructured region in hcGAS, and these acetylation events led to increased cGAS binding with DNA to promote cGAS activation in response to DNA challenge.^126^ Therefore, depending on the acetylation sites, acetylation of cGAS could either positively or negatively regulate cGAS activation and it is possible that acetylation occurring at different stages of infection may have a distinct function in cGAS activity control.

Notably, a recent proteomics effort in examining cGAS post-translational modifications upon HSV-1 infection from both human primary fibroblasts and HEK293T cells revealed new PTMs occurring on cGAS, including phosphorylation at Ser37, Ser116, Ser201, Ser221, Ser263, and acetylation at Lys198, Lys285, Lys355, and Lys414 in hcGAS.^127^ Further functional validation suggests that acetylation at Lys414 suppresses, while acetylation at Lys198 promotes hcGAS activation. Interestingly, hcGAS-Lys198 acetylation was found to be decreased by quantitative proteomics upon infection by either HSV-1 or HCMV (human cytomegalovirus), suggesting that these DNA viruses might hijack this acetylation regulation to targetedly inactivate cGAS to evade innate-immune surveillance.^127^ The detailed mechanism(s) mediating acetylation-dependent cGAS activity control on these sites remain unclear.

Taken together, a variety of post-translational modifications have been identified in regulating cGAS activity and function, including phosphorylation, acetylation, ubiquitination, and SUMOylation. Some modifications such as hcGAS acetylation at Lys384/Lys394/Lys414 occur at the resting state as a mechanism to ensure cGAS remains at a low activity until encountering DNA challenges. Some other modifications are triggered upon DNA challenge as means to acutely activate cGAS to ensure timely DNA sensing such as monoubiquitination of mcGAS-K335 or K27-linked polyubiquitination of mcGAS-Lys173/Lys384 that facilitates DNA binding and cGAS dimer formation. AKT and CDK1-mediated cGAS phosphorylation and inactivation of cGAS may serve as a mechanism to ensure cGAS is not aberrantly activated in cell cycle phases with exposures to naked DNA, including S/G2 and M phases. Thus, different modifications orchestra to fine-tune cGAS activation under distinct conditions and a tight control of cGAS activity through these modifications are crucial to maintain a proper innate-immune response. It is not surprising to find these modifications cross-talk with each other; however, given most of the studies focus on only one type of modifications, how tempo- and spatially these cGAS modifications occur and are regulated remain to be determined.

### Regulation of cGAS activation by cGAS-binding proteins

In addition to post-translational modifications directly occurring on cGAS proteins, various cGAS-binding partners, including both host proteins and viral/bacterial proteins, have been identified to modulate cGAS activation in order to regulate the innate-immune response. Generally, binding of host proteins to cGAS can either enhance cGAS activation to facilitate detection and clearance of cytosolic DNA derived from viral/bacterial infection (foreign-DNA) and damaged genome or mitochondrial DNA (self-DNA), or restrain cGAS activity at resting state to avoid unnecessary cGAS activation; while viral/bacterial proteins usually bind and inactivate cGAS to escape from innate-immune surveillance. The regulatory function of host proteins in facilitating cGAS activation can be due to their ability to bind cGAS to enhance DNA binding or bind DNA to bridge DNA for cGAS binding (Table 1).

**Table 1:** Summary of the regulatory mechanism of cGAS activation by ions, proteins, and small molecules

Type of regulators	Regulatory mechanism	Molecules involved	Regulatory function and effects	References
*cGAS activation*				
Ions	Physiological buffer	Zn^2+^, Mn^2+^, and Co^2+^	Augments cGAS enzymatic activity and sensitizes its binding to dsDNA	^43,163,164,223^
Proteins	Protein–protein interaction	G3BP1	Increases DNA-binding affinity	^128,129^
		PQBP1	Enhances cGAMP production	^131^
		TRIM21	Induces the ability of cGAS to detect the genomes of infectious viruses	^133^
		ZCCHC3	Enhances cGAS oligomerization	^132^
		HDAC3	Positively regulates the transcription of cGAS by deacetylating p65 at K122	^71^
		PCBP1	Directly interacts with reverse-transcribed HIV-1 ssDNA for its sensing by cGAS	^130^
		Streptavidin	Increases DNA binding	^157^
*cGAS inhibition*				
MicroRNAs	Transcription	miR-93 and miR-25	Negatively regulates cGAS expression in the immunosuppressive tumor microenvironment	^70^
Ions	Physiological buffer	K^+^ efflux	Attenuates cGAS activation	^166^
Proteins	Protein–protein interaction	Beclin-1	Inhibits the synthesis of cGAMP	^62^
		SAMHD1	Suppresses cGAS activity and limits innate-immune responses	^135,224^
		KSHV (LANA and ORF52)	Decreases cGAS enzymatic activity	^145,146^
		OASL	Restrains activation of cGAS and limits type I IFN production during DNA virus infection	^134^
		TRIM14	Attenuates DNA sensing during macrophage infection with *M. tuberculosis*	^96^
		HCMV (UL31, UL42, and UL83)	Decreases cGAS enzymatic activity and inhibits cGAMP production	^147–149^
		HSV-1(VP22, UL37, and UL41)	Suppresses the enzymatic activity of cGAS	^74,150,151^
		BAF	Restrains the formation of DNA-cGAS complexes and negatively regulates the activity of cGAS	^137^
		Nucleosome	The acidic path in H2A/H2B binds the Arg anchors in cGAS to suppress DNA binding	^139–144^
Proteases	Cleavage	Caspase 1/4/5/11	Cleaves and inactivates the cGAS protein	^156,160,161^

#### Host proteins binding to cGAS in promoting cGAS activation

For example, the host protein G3BP1 (GTPase-activating protein SH3 domain-binding protein 1) binds cGAS to potentiate cGAS binding with DNA, formation of cGAS oligomers, and activation of cGAS.^128,129^ Moreover, PCBP1 (Poly(rC)-binding protein 1) as a member of the heterogeneous nuclear ribonucleoprotein family, was reported to directly interact with cGAS in a viral infection-dependent manner to enhance cGAS binding to viral DNA, thus elevating cGAS activity.^130^ PQBP1 (polyglutamine binding protein 1) directly bridges reversely transcribed HIV-1 DNA and cGAS to trigger cGAS activation upon HIV infection in DCs (dendritic cells).^131^ ZCCHC3 directly interacts with dsDNA, leading to increased binding of dsDNA to cGAS and subsequent cGAS activation following viral infection.^132^ In addition, TRIM21 promotes proteasomal destruction of antibody-opsonized virions, leading to exposure of viral genome for cGAS detection and activation, thus indirectly facilitating cGAS activation.^133^

#### Host proteins binding to DNA in suppressing cGAS activation

The autophagy protein Beclin-1 directly interacted with cGAS and inhibited the synthesis of cGAMP by negatively regulating cGAS activity, thus increasing the autophagy-mediated degradation of cytosolic bacterial DNA to mediate innate antimicrobial immune response.^62^ Oligoadenylate synthetase family protein OASL directly bound to cGAS independent of dsDNA to non-competitively suppress cGAMP synthesis and subsequent type I IFN production during infection by DNA viruses such as vaccinia, herpes simplex, and adenovirus.^134^ Recently, it was reported that TRIM14 directly bound cGAS and acted as a scaffold protein between TBK1 and STAT3 to facilitate STAT3 phosphorylation in order to turn off STAT3 signaling and ISG expression during infection by *M. tuberculosis*.^96^ SAMHD1 has been observed as a restriction factor for HIV-1 infection by suppressing cGAS activity to limit innate and adaptive immune responses.^135^ Mechanistically, SAMHD1 facilitates clearance of naked DNA at stalled replication forks.^136^ In SAMHD1-deficient cells, cytosolic DNA accumulates to trigger the production of interferons, and this depends on MRE11 and RECQ1.^136^ Considering that SAMHD1 is frequently mutated in AGS and some cancer, it is plausible that inhibition of MRE11 and/or RECQ1 would attenuate cGAS activation through reducing the production of DNA fragments.

Sensing self-DNA should be tightly inhibited to avoid hyperactivation of innate-immune signaling leading to autoimmune diseases. Although a large portion of cGAS is present in the nucleus, its activation is suppressed by at least two mechanisms contributed by cGAS binding proteins. The first mechanism for inhibiting nuclear cGAS activation is achieved by binding cGAS to the chromatin-binding protein BAF (barrier-to-autointegration factor 1). As a natural opponent of cGAS activation, BAF binding to cGAS competes with DNA binding, thus restraining cGAS in an inactive state.^137^ The second mechanism is achieved by tight tethering of cGAS to chromatin,^138^ which is mediated by interactions between cGAS Arg anchors and H2A/H2B acidic patch residues, through which nucleosome binding to cGAS restrains cGAS binding to DNA for cGAS activation.^139–144^ In addition to cGAS binding proteins in restraining cGAS activation in the nucleus, cGAS phosphorylation by AKT and CDK1 also play a role in suppressing cGAS activation during cell cycle progression as discussed in the last section.

#### Viral/bacterial proteins binding to cGAS in modulating cGAS activation

Given to the essential function of cGAS in sensing infection by DNA viruses, RNA viruses (such as HIV-1, dengue, Zika, CHIKV, and others, detailed below), and bacteria, these human pathogens evolve mechanisms to disable cGAS cytosolic DNA sensor function. One of such mechanisms includes utilizing viral/bacterial proteins to directly bind cGAS to suppress cGAS activation. For example, the cytoplasmic isoforms of LANA (latency-associated nuclear antigen) from Kaposi sarcoma herpesvirus (KSHV) were identified as a cytosolic cGAS binding partner, and LANA binding to cGAS inhibited cGAS activation and subsequent phosphorylation of TBK1 and IRF3 that induces KSHV reactivation from latency.^145^ However, how LANA binding restrains cGAS activation remains to be determined. In addition, ORF52 is a gamma-herpesvirus tegument protein and was found to interact with both cGAS and DNA to suppress the antiviral immune response.^146^ Although ORF52 binding to cGAS does not affect cGAS binding to DNA, ORF52 binding to the cGAS/DNA complex impeded cGAS activation, presumably through blocking proper cGAS protein conformational changes necessary for its enzymatic activation. In addition, three human cytomegalovirus (HCMV) tegument proteins were found to bind cGAS and modulate cGAS activation. Specifically, UL31 was reported to bind cGAS to dissociate DNA binding, therefore leading to reduced cGAS activation for innate-immune evasion.^147^ Similarly, UL42 bound to both cGAS and STING, and UL42 binding to cGAS suppressed DNA binding, cGAS oligomerization, and cGAS enzymatic activity.^148^ UL83 only interacted with cGAS but not STING, and binding of UL83 to cGAS impeded cGAS activation upon HCMV infection, while how UL83 inhibits cGAS function remains unclear.^149^ Moreover, three HSV-1 (herpes simplex virus 1) tegument proteins were also observed to bind cGAS in suppressing cGAS activation. UL37 is an HSV-1 tegument protein and a deaminase bound to cGAS to deamidate hcGAS on Asn210, Asn389, Gln451, and Gln454 residues, leading to a suppression of intrinsic cGAS enzymatic activity without affecting cGAS binding to DNA, cGAS dimerization or substrate nucleotides.^150^ Another HSV-1 protein UL41 utilized its endoribonuclease activity to degrade cGAS mRNA, through downregulating cGAS protein expression to suppress innate-immune sensing.^74^ VP22 was also found to bind and suppress cGAS activation upon HSV-1 infection, while the detailed molecular mechanism for this suppression remains to be determined.^151^

F17, a protein encoded by poxviruses, exerted a crucial role in evading host antiviral responses by promoting cGAS degradation through elevating mTORC2 activity by sequestering Raptor and/or Rictor from mTORC1 during infection.^152^ The mammalian nuclear protein NONO directly bound HIV capsid in the nucleus to facilitate cGAS association with HIV DNA, thus playing an indispensable role in promoting cGAS-mediated innate-immune activation upon HIV infection.^153^ In addition, the DS2B protease cofactor from an RNA DENV virus (dengue virus) targeted cGAS for lysosomal-dependent degradation to evade cGAS detection of damaged/leaked mitochondrial DNA upon DENV infection.^154^ Similarly, the CHIKV (Chikungunya virus) capsid protein suppressed transcription of IFN-β during CHIKV infection through inducing autophagy-dependent cGAS degradation.^155^ Furthermore, the NSP1 protein from the RNA virus Zika stabilized caspase 1 to enhance cGAS cleavage and inactivation, leading to evasion of innate-immune sensing.^156^ Together, these viral proteins through binding cGAS to guide cGAS for degradation (through proteasome, lysosome, and autophagy) or cleavage as an approach to facilitate evasion of host detection. If any bacterial protein plays a similar function remains unknown.

In addition to viral proteins, recently our group identified a bacterial protein streptavidin in directly binding and activating cGAS through promoting cGAS association with DNA and subsequent cGAS liquid phase transition and enzymatic activation.^157^ Given that streptavidin is currently widely used as a tool in biotechnology to monitor biotin-tagged molecules, and a drug delivery vehicle with immune adjuvant activity in clinic, our studies horn an alarm to use streptavidin in these settings with caution due to its ability in facilitating activation of innate immunity.

### Regulation of cGAS activation by cGAS proteolytic cleavage

Proteolytic cleavages by protein proteases play key roles in regulating protein structure and function, especially targeted and limited proteolysis^158^ including activation of growth factors such as TGFβ by proteases^159^. Caspase 1 cleaves Asp140 and Asp157 residues,^160^ while caspase 3 cleaves the Asp319 residue^161^ in hcGAS to restrain cGAS activation upon DNA or RNA viral infection. Caspase 4, 5, and 11 activated during noncanonical inflammasome formation were also shown to be able to cleave cGAS in the cGAS N-terminal R/K rich region to facilitate viral infection by inactivating cGAS sensing.^160^ In addition, caspase 3/7 or caspase 9 has also been shown to cleave cGAS at unknown residues to suppress mtDNA-induced cGAS activation and IFN production.^162^ Interestingly, the Zika virus NSP1 protein stabilized caspase 1 to enhance cGAS cleavage and inactivation, leading to evasion of innate-immune sensing,^156^ highlighting the pathophysiological significance of cGAS proteolytic cleavage in facilitating viral infection. In addition to viral infection, if cGAS cleavage by caspases occurs in physiological conditions or developmental stages, and if cGAS can be cleaved by non-caspase proteases remain interesting questions to answer.

## Regulation of cGAS activation by ions

Interestingly, it has been observed that the cGAS enzyme activity was different between physiological buffers and low-salt buffers, and this difference was caused by ions present in buffers.^43^ Specifically, similar to many other enzymes, the inclusion of Zn^2+^, Mn^2+^, or Co^2+^ ions in physiological buffers facilitated cGAS activation.^43^ The reason for Zn^2+^ in promoting cGAS activation is largely due to the fact that Zn^2+^ induces cGAS phase transition in the presence of DNA.^43^ In addition, Mn^2+^ could also augment cGAS enzymatic activity by enhancing its sensitivity to low doses of dsDNA usually nonstimulatory,^163^ or directly activate cGAS independent of dsDNA to synthesize cGAMP,^164^ presumably through activating monomeric cGAS without dsDNA.^165^ Notably, there is new evidence demonstrating that K^+^ efflux might suppress cGAS activation in decreasing the type I interferon responses,^166^ while the underlying mechanistic insights warrant further investigations. Interestingly, some copper (Cu^2+^) complexes used in anticancer therapy inhibit topoisomerase 1 and 2, leading to DNA breaks and DNA fragments to activate cGAS signaling.^167^ Notably, transporters for these ions have been observed dysregulated in cancer,^168^ and it is plausible altered ion transport in cancer may facilitate cancer evasion of immune surveillance by modulating cGAS signaling. Although there is no direct evidence to support this speculation, it definitely warrants further investigations.

## Regulation of cGAS activity by small molecules

Given the essential function of cGAS in cytosolic DNA sensing and various pathological conditions including both autoimmune diseases and cancer, research efforts have also been devoted to developing small molecules that modulate cGAS activity. RU.521 has been developed as a cGAS inhibitor by competitive binding to cGAS catalytic pocket with cGAS substrates ATP/GTP.^169^ Through screening a compound library using a luciferase-based platform, G140/G150 was found as potent cGAS inhibitors.^170^ In another fluorescence polarization assay utilizing recombinant active human cGAS proteins in vitro, PF-06928215 was characterized to be able to inhibit cGAS activity.^171^ Interestingly, a subsequent virtual compound screen based on a structure derived from PF-06928215 binding to cGAS led to the identification of additional compounds S2 and S3 as potent cGAS inhibitors.^172^ A virtual drug screen combined with further medicinal chemistry studies based on a solved structure of mouse cGAS led to the development of Cu-32 and Cu-76 as cGAS inhibitors by binding to cGAS DNA-binding pocket to prevent cGAS dimerization and subsequence activation.^173^ Through establishing compounds that stereo-chemically distinct from traditional compounds, SI-56 was found to be able to inhibit cGAS-induced IFN production in cells.^174^ In addition to these newly identified compounds, a handful of previously commercially available compounds have also been found to suppress cGAS activity. This includes hydroxychloroquine (HQC),^175^ X6 (used to treat malaria),^176^ and suramin^177^ that block cGAS binding to DNA, as well as aspirin^125^ that inhibits cGAS activity through directly acetylating cGAS proteins. Notably, to date, there is no cGAS activator that has been successfully developed.

## Regulation of cGAS activation by lipids

The N-terminal unstructured region in cGAS has been shown to be necessary for DNA binding. A recent study uncovered a novel role of this N-terminus in directly binding phosphoinositide that positions cGAS at the plasma membrane.^47^ This ensures the resting state of cGAS is suppressed and separated from detecting cytosolic DNA, serving as an additional layer of regulatory mechanism.

## Regulation of the cGAS enzymatic product 2′3′-cGAMP

2′3′-cGAMP, a special cyclic-dinucleotide (CDN) was characterized as the major cGAS enzymatic product.^27^ Cyclic dinucleotides are conserved secondary messengers in both prokaryotes and eukaryotes. In the former, CDNs regulate various cellular processes while in latter CDNs largely activate innate-immune response.^178^ Cyclic di-GMP and cyclic di-AMP are second messengers in gram-negative and gram-positive bacteria, respectively. Asymmetric cyclic AMP-GMP was identified in *Vibrio cholerae* as a virulence signaling molecule, synthesized by DncV, a dinucleotide cyclase with sequence similarity to eukaryotic OSA1 (oligoadenylate synthetase).^179^ The cGAS enzymatic product 2′3′-cGAMP was synthesized from GTP and ATP, with AMP-2′-GTP^180^ or 5′-pppGpG^169^ as intermediates. Although these intermediate products have been observed, how long they can stay in cells and if any of these intermediates exerts physiological function remains unknown.

As a potent STING agonist, 2′3′-cGAMP displays an exciting function in triggering innate immunity to facilitate anticancer treatment through promoting tumor-infiltrating T cells. Administration of 2′3′-cGAMP directly into mice showed a synergy with anti-immune-checkpoint blockade (anti-PD-L1) in suppressing melanoma growth.^68^ Although asymmetric, 2′3′-cGAMP was efficiently transferred inside or outside of cells, which was largely mediated by various characterized cGAMP importer SLC19A1^181,182^ or transporter LRRC8A:C/E heteromeric channels.^183,184^ Increased expression of SLC19A1 was observed in SLE (systemic lupus erythematosus) patients^185^ that might promote innate immunity activation. 2′3′-cGAMP was found to be steadily released from cells^186^ but degraded by extracellular ENPP1 (ecto-nucleotide pyrophosphatase/phosphodiesterase).^187^ Similar to 2′3′-cGAMP administration, inhibiting ENPP1 that leads to 2′3′-cGAMP accumulation synergized with IR (ionizing radiation) to impede tumor growth.^186^ Interestingly, 2′3′-cGAMP was reported to exert a bystander activity that cGAMP producing cells can inter-cellularly transfer cGAMP into bystander cells that rapidly amplify antiviral immunity signals^188^ to enhance antiviral immunity.^183^ 2′3′-cGAMP could also be transferred from epithelial cells into co-cultured macrophages to transactivate STING signaling in a connexin-dependent manner.^189^ On the other hand, brain metastatic cancer cells could utilize the carcinoma-astrocyte gap junctions to transfer 2′3′-cGAMP into astrocytes, where 2′3′-cGAMP promotes inflammatory cytokine secretion as paracrine signaling to fuel tumor growth.^190^ Thus, there are both problems and benefits for 2′3′-cGAMP in regulating innate immunity and anticancer immunity. Although ENPP1 was identified as the major enzyme degrading extracellular 2′3′-cGAMP, the identities of the host-specific intracellular 2′3′-cGAMP degrading enzyme(s) remain unknown. Nonetheless, recently viral and metazoan poxins (poxvirus immune nucleases) were reported to cleave intracellular 2′3′-cGAMP (into linear Gp[2’-5’]Ap[3’]) to restrain STING activation as an approach to evade innate-immune detection.^191^

## Roles of cGAS in human autoimmune and inflammation diseases

cGAS/STING innate-immune signaling plays a crucial role in many inflammation-related diseases, such as cardiovascular disease, neurodegenerative disease, inflammatory bowel disease, diabetes, fibrosis, lupus, arthritis, and psoriasis.^192^ (Table 2) For example, in Alzheimer’s disease (AD)-associated cardiac dysfunction, mutations of AD genes PS1 (presenilin 1), and PS2 suppressed mitophagy and contractile function through downregulating the cGAS/STING signaling.^193^

**Table 2:** Human diseases related to deregulated cGAS signaling

Disease	Possible pathogens involved	Regulatory function and effects	References
Acute pancreatitis (AP)	Unknown	*cGAS* loss-mediated reduction in IFN-β release exerts a protective role in AP	^196^
Age-dependent macular degeneration (AMD)	Mitochondrial DNA (mtDNA)	mtDNA driven cGAS activation to potentiate type I IFN signaling	^200^
Alcoholic liver disease (ALD)	Unknown	cGAS activation observed in both ALD mouse models and ALD patients, and cGAS activation correlates with disease severity	^225^
Aicardi–Goutières syndrome (AGS)	Nuclear DNA	Increased expression of type I IFNs and interferon-stimulated genes (ISG) caused by cGAS activation	^219^
Aujeszky’s disease	Nuclear DNA	Activation of cGAS-mediated innate immunity observed in disease to increase host resistance to viral infection	^220^
Alzheimer’s disease	mtDNA	mtDNA activates cGAS signaling and mitophagy via an ALDH2-dependent mechanism	^193^
Asthma	Cytosolic dsDNA	*cGAS* deletion in mouse airway ECs significantly attenuated OVA- or HDM-induced airway eosinophilic inflammation, mucus overproduction, and airway hyperresponsiveness (AHR)	^198^
Bloom syndrome	Unknown	Elevated ISG expression observed in peripheral blood presumably due to cGAS activation	^226^
Familial chilblain lupus (FCL)	Cytosolic DNA	Aberrant IFN signature and inflammasome activation observed presumably due to cGAS activation	^65,227^
Hutchinson–Gilford progeria syndrome	Unknown	Activation of cGAS and a robust STAT1-regulated IFN-like response	^228^
Huntington disease (HD)	Unknown	cGAS is activated in HD in mediating inflammatory and autophagy responses	^229^
Myocardial infarction (MI)	Extracellular DNA	During cardiac ischemia, cGAS serves as a pattern-recognition receptor in the sterile immune response	^202,230^
Parkinson’s disease	mtDNA	*LRRK2* deletion in mice causes mitochondria stress leading to chronic cGAS activation to produce IFNs.	^231^
Systemic lupus erythematosus (SLE)	Cytosolic DNA	Caused by increased cGAS activation due to aberrant accumulation of cytosolic DNA	^65,66,195^
Cancer	Cytosolic DNA	Reduced expression of cGAS associated with poor patient survival in lung, brain, colorectal, and breast cancer patients and creates a tumor-prone immune microenvironment through suppressing innate immunity; on the other hand, cGAS promotes genome instability through inhibiting homologous recombination to promote lung cancer growth and 2'3'-cGAMP facilitates breast tumor metastasis in brain.	^64,68,70,207^

In ALS (amyotrophic lateral sclerosis), cytoplasmic accumulation of the nuclear DNA/RNA binding protein TDP-43 was reported to trigger mtDNA release through mPTP (mitochondrial permeability transition pore) that subsequently activates cGAS/STING signaling to induce neuroinflammation through activating NF-κB and promoting type I interferon (IFN) production.^194^ Strikingly, in mouse models, *cGAS* deletion greatly rescued autoimmune-disease phenotypes caused by *Trex-1* loss such as AGS^65^ and SLE.^66^ These observations strongly support a critical role of cGAS signaling in contributing to autoimmune diseases and advocate for applying cGAS inhibitors in alleviating these disease symptoms in clinic. Hyperactivation of cGAS/STING signaling followed by an overproduction of harmful pro-inflammatory cytokines contributed to the pathogenesis of not only autoimmune diseases^59,66,195^ but also acute pancreatitis^196^ and insulin resistance.^197^ In addition, emerging evidence suggests that cGAS also promoted TH2 allergic inflammation likely via regulating airway epithelial GM-CSF production and might play an important role in immune responses of asthma pathophysiology.^198^ In mouse models, deletion of *cGAS* in airway epithelial cells reduced allergic airway inflammation.^198^ In another study, activation of cGAS signaling by mtDNA was observed to prohibit the YAP-mediated endothelial cell proliferation program to promote inflammatory injury.^199^

In addition, several studies have revealed that cGAS activation by self-DNA derived from chromatin instability (CIN), damaged mitochondria, micronuclei, or cell debris was connected to more familial and complex diseases, including myocardial infarction (MI) and age-related macular degeneration (AMD).^57^ MI has a high fatality rate in human, concomitant with increased inflammation and immune responses. This disease is caused by massively increased type I IFN production from heart macrophages caused by hyperactivation of cGAS–STING signaling, leading to massive death of cardiomyocytes. The importance of cGAS activation in this disease is also supported by the observation that knockout of *cGAS*, *IRF3*, and *IFNAR1* in MI-related mouse models remarkably increased early survival.^57,200^ In mice receiving TAC (transverse aortic constriction) to induce heart failure or sham operation, downregulation of cGAS was found to be able to improve early survival, maintain left ventricular contractile function and attenuate cardiac hypertrophy or apoptosis induced by TAC, presumably through reducing inflammatory cytokine production.^201^ In addition, the ischemic myocardial injury was reported to activate cGAS signaling by inducing the release of nucleic acids that subsequently trigger inflammatory programs to promote macrophage transformation and regulate post-injury cardiac repair.^202^ In addition, cGAS signaling may also be indispensable for radiation-induced cardiovascular diseases given that radiation-induced DNA damage activates cGAS signaling and induces inflammation.^203^ Due to the fact that the heart is an organ relying on ATP consumption with abundant mitochondria, mitochondria dysfunction (leading to leakage of mtDNA and increased mtROS) triggers cGAS activation that contributes to cardiac inflammation.^204^ Thus, suppression of cGAS/STING signaling might serve as a therapeutic direction for treating these inflammation-related heart diseases through reducing inflammation signals.

## Roles of cGAS in cancer

The cGAS signaling also plays important roles in regulating tumor immunity and tumor oncogenicity through modulating both tumor immune microenvironment and intrinsic tumorigenesis programs (such as cell senescence and DNA damage response). First, cGAS signaling is indispensable for the establishment of an anti-tumor immune environment given that type I IFNs stimulated by cGAS signaling bridge innate and adaptive immunity. Specifically, the tumorigenesis process is usually accompanied by DNA damage and leakage of damaged genomic or mitochondrial DNA in cancer cells or uptake of damaged cancer cells by dendritic cells (DCs).^205^ These increased levels of cytosolic DNA activate cGAS signaling to promote IFN production in cancer cells to prime tumor-specific T-cell infiltrations, and in DCs to lead to DC maturation.^206^ Subsequently, mature dendritic cells present tumor-associated antigens to activate CD8 + T cells in order to eradicate cancer cells through the immune system. In lung adenocarcinoma patients, decreased cGAS activity has been observed associated with poor patient survival by bioinformatics analyses;^207^ while in a recent study overexpression of cGAS has been observed to promote lung tumor growth through inhibiting homologous recombination.^64^ Consistent with a tumor-suppressive role of cGAS, expression of two hypoxia-responsive microRNAs miR-25 and miR-93 that suppressed cGAS activity through downregulating cGAS expression has been observed significantly increased in cohorts of glioma, colorectal carcinoma, and breast carcinoma patients accompanied by poor prognosis.^70^ Consistent with this notion, cGAMP administration synergized with anti-PD-L1 immune checkpoint blockade to suppress melanoma growth in a mouse model, through boosting innate immunity.^68^ Blocking extracellular cGAMP degradation by ENPP1 inhibitors also synergized with radiation to induce anti-tumor immunity.^186^ Recently, in tumors with dMMR (DNA mismatch repair deficiency) with increased neoantigen loads, cGAS signaling in sensing cytosolic DNA was shown to be indispensable for an effective response to anti-PD-1 therapy.^203^ Mechanistically, loss of the MutLα subunit MLH1 observed in half of dMMR cancer triggered the accumulation of cytosolic DNA due to loss of MLH1-dependent control of Exo1 (exonuclease 1) in DNA repair, leading to increased DNA excision by Exo1.^204^ In addition to modulating tumor immune environment, cGAS activation has been also observed to modulate intrinsic cellular programs. For example, cGAS was observed to be indispensable to antagonize cellular senescence, and *cGAS* loss facilitated the primary cell immortalization process.^55^

On the other hand, cGAMP transfer seems to promote brain metastasis. Specifically, the protein connexin 43 and proto-cadherin 7 induced cGAMP transfer from cancer cells migrated to and colonized in the brain to astrocytes via gap intersections. This promoted NF-κB signaling activation and production of inflammatory cytokines in astrocytes, which could function in a paracrine manner to fuel cancer cell growth to increase brain metastasis.^208^ In another study, chromosomal instability (CIN) that is commonly observed in cancer was observed to promote tumor metastasis to distal organs through generating cytosolic DNA, activating cGAS signaling, and subsequent noncanonical NF-κB activation.^209^ Notably, the extracellular cGAMP hydrolysis enzyme ENPP1 was found to be able to promote tumor metastasis by degrading cGAMP into adenosine to facilitate tumor immune evasion, and ENPP1 inhibition increased responses to immune checkpoint blockade.^210^ Thus, it seems cGAMP may exert distinct function in cancer and bystander cells in a cellular context-dependent manner. If and how cGAS signaling in cancer and host cells communicate to modulate both intrinsic cellular programs and tumor microenvironment to modulate cancer growth and metastasis warrant further in-depth investigations.

## Concluding remarks and future perspectives

As a key component of PRRs (PAMP and DAMP) related host defense system through specifically sensing cytosolic DNA, since its discovery in 2013, cGAS has drawn extensive attention from the research community, not only due to its canonical roles in governing innate immunity^211^ and novel function in directly regulating cellular processes such as DNA damage^64^ but also its potential as a drug target in treating both autoimmune diseases and cancer. On one hand, cGAS signaling is indispensable to sense invasive cytosolic DNA to clear viral/bacterial infection. Cancer also hijacks a variety of mechanisms to inactivate cGAS signaling to evade immune surveillance. As a result, activating cGAS signaling, or administration of cGAMP benefits cancer treatment.^68^ On the other hand, hyperactivation of cGAS signaling leads to various human autoimmune diseases such as SLE and AGS, which can be treated by cGAS inhibition.^65,66^ Thus, both cGAS agonists and antagonists might be therapeutic directions for treating cancer and autoimmune diseases, respectively.

Analyses of the evolution route of cGAS suggest that DNA sensing and cGAMP synthesis are not only restricted in vertebrates. Recent studies identified cGAMP-synthesizing enzymes in non-vertebrate species such as bacteria^212^ and sea anemone species *Nematostella vectensi*.^213^ Bacterial cGAS-like enzymes could synthesize a variety of cyclic-dinucleotides that play important roles in anti-phage defense.^214^ cGAMP production was also observed in bacteria.^179^ In Gram-negative bacteria *Virbrio cholerae* 3’3’-cGAMP synthesized by DncV activated a phospholipase CapV (cGAMP-activated phospholipase in Vibrio) to cause degradation of the bacterial inner membrane.^215^ Thus, it seems cGAS/cGAMP/STING signaling is an evolutionarily conserved defense system with an ancient evolutionary root. Among vertebrates, the essential function of cGAS in sensing cytosolic DNA to produce type I IFN was more studied in birds and mammals,^216^ which might be due to the presence of common pathogens in these species that shapes up similar selection pressure for cGAS evolution.^217^ It remains unclear but interesting to explore if there are other cGAS-like enzymes in vertebrates beyond mammals and birds that play essential roles in sensing cytosolic DNA, and what is the function of ancestral cGAS homologs.

Given that both overphysiologically reduced and increased cGAS activation may cause human diseases, cGAS activation is tightly controlled spatially and temporally at multiple levels. This includes acute responses by post-translational modifications of cGAS and cGAS binding proteins and relatively long-term responses by cGAS transcriptional regulations. As a rapidly developing research field, there are still many interesting questions that remain to be answered. For example, if there are cross-talks among different cGAS PTMs, if certain PTMs could be recognized by cGAS binding partners and if cytosolic and nuclear cGAS are differentially modified and recognized by different binding partners. These multiple cGAS regulatory PTMs also raise a crucial question that how these cGAS PTMs acutely and accurately respond to infection in a coordinated manner at both spatial and temporal aspects. Furthermore, the tight control of cGAS activation is also achieved by restraining cGAS in certain cellular compartments (e.g., restraining cGAS on the plasma membrane through binding PI(4,5)P_2_^218^ or in the nucleus by association with nucleosomes/BAF), in a cell cycle-dependent manner by AKT and CDK1, and fine tuning cGAS activation by ions. Considering that dysregulation in suppressing cGAS sensing of nuclear DNA leads to Aicardi–Goutières syndrome (AGS)^219^ and Aujeszky’s disease,^220^ it is plausible that genetic alternations on cGAS or cGAS regulatory proteins contribute to pathological conditions. These complex and integrated signaling pathway analyses rely on more systematic approaches to tackle in the future.

Small molecules to inhibit cGAS activation have been successfully developed in treating autoimmune diseases, however, side-effects in suppressing the immune microenvironment that favors tumorigenesis would need to be taken into consideration. On the other hand, there is no cGAS activators have been identified that might be due to the vital role of DNA in cGAS activation. Nevertheless, administration of cGAMP, the major cGAS enzymatic product has shown effects in restoring immune-friendly microenvironment in facilitating anti-immune checkpoint blockage therapies^68^ and increasing concentrations of extracellular cGAMP levels through inhibiting cGAMP degrading enzyme ENPP1 has been reported to suppress tumor metastasis.^210^ Due to the cost for cGAMP synthesis, as well as the concern that cGAMP is actively degraded by extracellular protease ENPP1,^187^ small-molecule cGAS activators, together with ENPP1 inhibitors, would be a more cost-effective and robust approach as an adjuvant to convert “immune-cold” tumors into “immune-hot” tumors by inducing effective CD8 + T-cell infiltrates.

In addition to canonical cGAS function in sensing cytosolic DNA to initiate innate immunity, cGAS has also been observed to exert physiological function in regulating intrinsic cellular processes such as triggering senescence,^55,56^ modulating DNA damage responses through binding DNA damage repair factors^57,64,221^ and suppressing genomic instability through decelerating replication forks.^222^ In the past 8 years since the discovery of cGAS as a cytosolic DNA sensor, many researchers have dedicated efforts in elucidating cGAS regulations and functions through genetic, biochemical, and biophysical aspects that laid a solid foundation for future studies (Fig. 1). To this end, identification and characterization of the additional pathophysiological functions of cGAS would further advance our understanding of cGAS biology, and provide insights into new treatment modalities targeting cGAS signaling for human diseases.
